# Gastrointestinal Microbiota in Gastric Cancer: Potential Mechanisms and Clinical Applications—A Literature Review

**DOI:** 10.3390/cancers16203547

**Published:** 2024-10-21

**Authors:** Mengjiao Wu, Chenjun Tian, Zhenwei Zou, Min Jin, Hongli Liu

**Affiliations:** 1 Cancer Center, Union Hospital, Tongji Medical College, Huazhong University of Science and Technology, Wuhan 430022, China; wumengjiaos@163.com (M.W.); zouzhenwei@hust.edu.cn (Z.Z.); 2 Hubei Key Laboratory of Precision Radiation Oncology, Wuhan 430022, China; 3 Institute of Radiation Oncology, Union Hospital, Tongji Medical College, Huazhong University of Science and Technology, Wuhan 430022, China; 4 The First Clinical Medical College, Lanzhou University, Lanzhou 730000, China; 18627935503@163.com; 5 The Eighth Hospital of Wuhan, Wuhan 430012, China

**Keywords:** gastrointestinal microbiota, gastric cancer, *Helicobacter pylori*, microbiome remodeling, therapeutic strategies

## Abstract

**Simple Summary:**

The gut microbiota significantly influences gastric cancer development, with *H. pylori* being a primary risk factor. Other microbes also contribute through chronic inflammation, genotoxic effects, and metabolic changes. Alterations in microbiota can impact the efficacy and side effects of cancer therapies. New microbiome-targeted treatments, including dietary changes, antibiotics, probiotics, synbiotics, and fecal transplants, show potential for improving therapeutic outcomes and reducing side effects. Understanding the microbiota’s role in gastric cancer could lead to more effective treatment strategies.

**Abstract:**

Emerging evidence highlights the crucial role of gastrointestinal microbiota in the pathogenesis of gastric cancer. *Helicobacter pylori* (*H. pylori*) infection stands out as a primary pathogenic factor. However, interventions such as anti-*H. pylori* therapy, gastric surgeries, immunotherapy, and chronic inflammation significantly remodel the gastric microbiome, implicating a broader spectrum of microorganisms in cancer development. These microbial populations can modulate gastric carcinogenesis through various mechanisms, including sustained chronic inflammation, bacterial genotoxins, alterations in short-chain fatty acids, elevated gastrointestinal bile acids, impaired mucus barrier function, and increased concentrations of N-nitrosamines and lactic acid. The dynamic changes in gut microbiota also critically influence the outcomes of anti-cancer therapies by modifying drug bioavailability and metabolism, thus affecting therapeutic efficacy and side effect profiles. Additionally, the effectiveness of radiotherapy can be significantly impacted by gut microbiota alterations. Novel therapeutic strategies targeting the microbiome, such as dietary interventions, probiotic and synbiotic supplementation, and fecal microbiota transplantation, are showing promise in cancer treatment. Understanding the intricate relationship between the gut microbiota and gastric cancer is essential for developing new, evidence-based approaches to the prevention and treatment of this malignancy.

## 1. Introduction

Gastric cancer (GC) is the fifth most common cancer and the third leading cause of cancer-related deaths worldwide. Despite advances in therapeutic strategies for GC, ranging from surgery, chemotherapy, radiotherapy, and molecularly targeted therapies to immunotherapy, mortality rates remain very high. Addressing the challenge of reducing mortality from GC has become an urgent public health issue. The development of GC involves a multifactorial and dynamic process that results from the interaction of various genetic and environmental factors within the host. GC presents as a cancer type characterized by a high degree of heterogeneity. The Cancer Genome Atlas (TCGA) classifies GC into four subtypes [1], including (i) ebvirus (EBV)-positive tumors, (ii) microsatellite instability (MSI) tumors, (iii) genome stable (GS) tumors, and (iv) chromosomal instability (CIN) tumors. According to Lauren’s classification, GC can be divided into two distinct types: intestinal and diffuse [2]. Intestinal-type GC is defined by the Correa cascade, which consists of a series of changes starting from normal gastric mucosa, erosive gastritis, atrophic gastritis (AG), and intestinal epithelial metaplasia (IM), leading to heterogeneous proliferation and eventual progression to GC in situ and invasive carcinoma [3]. Although the development of diffuse GC is poorly understood, it is generally believed that *Helicobacter pylori* (*H. pylori*) and inflammation play a potential role [4].

The gastrointestinal microbiota is a complex community of bacteria, archaea, fungi, and viruses present in the host’s digestive tract. The collective genetic material of the microbiota and the environmental conditions are collectively referred to as the microbiome. Although considered a “neglected organ” at the beginning of the 21st century [5], this view changed dramatically in 2018, with the recognition that the microbiome is an ecosystem of organisms that communicate extensively with other organ tissues of the host, with increasingly prominent impacts on health and disease. Studies have shown that gastrointestinal microbes, as well as metabolites, contribute to cancer development in a comprehensive and multifaceted manner, aid in metastasis, lead to chemotherapy resistance, and influence the efficacy of immunotherapy. *H. pylori* infection is considered a major risk factor for GC [4,6]. In recent years, with the development of sequencing platforms and macrogenomics, the potential role of enriched bacteria other than *H. pylori* in GC has received attention from researchers. A growing body of evidence emphasizes the strong correlation between the gastrointestinal microbiota and the initiation and progression of GC.

Despite extensive research, the exact composition of the gastrointestinal microbiota in GC remains poorly understood, and the mechanisms by which these microbial communities evolve during GC development are not fully elucidated. This paper explores the link between gastrointestinal microbiota dysbiosis and GC, emphasizing the potential of the gastric microbiota as both a biomarker and a target for clinical treatment strategies.

## 2. Gastrointestinal Flora Associated with GC

The stomach used to be considered a sterile organ due to the presence of gastric acid, a normal acidic environment that prevents most bacteria from colonizing the stomach [7]. It was not until the discovery of *H. pylori* in 1983 that this view was overturned [8]. In 2006, researchers confirmed for the first time by gene sequencing that the gastric flora has a diverse community of 128 phylotypes, including 5 dominant phyla, namely, *Anaplasma*, *Firmicutes*, *Clostridium*, *Actinobacteria*, and *Aspergillus* (including *H. pylori*). Of these, the gastric mucosa is dominated by the *Firmicutes* and *Aspergillus* phyla, whereas the gastric juice is most commonly colonized by the *Firmicutes*, *Clostridium*, and *Actinobacteria* phyla [9]. With the wide application of macrogenomics and high-throughput sequencing technologies in microbiology, the diversity and complexity of gastrointestinal microbial communities associated with GC have been gradually revealed.

### 2.1. H. pylori and GC

*H. pylori* is a recognized carcinogen closely related to the development of GC, and it is a microaerobic Gram-negative bacterium that parasitizes the epithelium of the human gastric mucosa [10]. *H. pylori* is not a native inhabitant of the stomach but is an exogenous microflora infected via the oral–oral or fecal–oral pathway [11]. Continuous exposure of the gastric mucosa to *H. pylori* infection can trigger an inflammatory cascade, leading to AG and significantly increasing the risk of GC. *H. pylori* infects approximately half of the world’s population, with a prevalence rate of 80% in developing countries [12]. The carcinogenicity of *H. pylori* is influenced by the virulence of the strain, the bacterial load, genetic predisposition, lifestyle habits of the host, and other microbiota factors. Early anti-*H. pylori* treatment and eradication of *H. pylori* can reduce the incidence of GC, which is more obvious in individuals without precancerous lesions of GC [13,14]. It is not clear that the eradication of *H. pylori* prevents GC by blocking a stage in the course of the infection. A consensus of experts in some countries recommends that *H. pylori* should be eradicated preferably before the chronic AG stage to prevent GC [15,16,17]. *H. pylori* prefers colonizing healthy gastric mucosa, and its abundance is notably reduced in tumor tissues compared to adjacent non-tumor tissues [18,19]. Yang et al. discovered a significant increase in *H. pylori* within the peritumoral microhabitat of patients with favorable prognoses [20]. As GC advances from conditions like gastritis or gastric ulcers, the presence of *H. pylori* colonization tends to decrease [21], eventually disappearing as GC progresses [22].

In the process of GC formation, gastric acid secretion gradually decreases [23], and *H. pylori* is often lost, leading to a relatively mild acid environment in the intestines of GC patients and the formation of a unique intestinal microbiota, which is favorable for the colonization of pathogenic bacteria [24]. On the other hand, treatment for *H. pylori* eradication could potentially result in heightened bacterial diversity [25]. Nakano et al.’s research found that compared with non-gastric cancer patients, the average abundance of unclassified *Oxalobacteraceae*, *Capnocytophaga*, and *Haemophilus* increased at the genus level in patients with GC treated with *H. pylori* eradication [26].

Significant differences in gastric microbiota exist between patients infected with *H. pylori* and those not infected with *H. pylori*, suggesting that *H. pylori* may play a role in other microbial dysbiosis [27,28,29,30]. Metagenomic data reveal that as the abundance of *H. pylori* decreases, the occurrence of other microbes increases [21]. In late-stage gastric cancer patients, a reverse expression relationship is observed between *H. pylori* and various bacteria, including spirochetes, Neisseria, Prevotella, Veillonella, and Rothia [30]. However, several other studies comparing the taxonomic composition of gut microbiota between *H. pylori*-positive and *H. pylori*-negative individuals did not find significant differences [9,31]. Ai et al. analyzed RNA-Seq data from 727 gastric cancer samples and found that *H. pylori* and Lysobacter were markedly more prevalent in normal tissues, whereas Pseudomonas was notably more prevalent in tumor tissues [32]. To date, conclusive evidence is lacking on whether *H. pylori* acts as a bacterial driver and interacts with other gastric bacteria. Future research should investigate the precise role of *H. pylori* within the gastric microbiota and its complex relationships with other microbes.

### 2.2. Bacteria Other than H. pylori and GC

With the application of second-generation sequencing technology in microbiology, besides *H. pylori*, many studies have also discovered other acid-resistant bacteria. Kim et al. utilized 16S ribosomal RNA gene analysis to study *H. pylori*-negative GC tissue and found an increase in the relative abundance of the phyla Actinobacteria, *Bacteroidetes*, and *Firmicutes* [33]. Maldonado-Contreras et al. and Kaisa et al. both reported higher levels of *Actinobacteria* in *H. pylori*-negative GC [31,34]. Park et al. discovered that gastric fluid samples from GC patients exhibited an increase in *Lactobacillus* and *Veillonella* while showing a decrease in *Verrucomicrobia* and *Deferribacteres* [35]. *Akkermansia*, a member of the phylum Verrucomicrobia, has been reported to be linked with GC progression [36]. Png et al. observed that the development of GC was characterized by an elevation in *Proteobacteria* and a reduction in *Bacteroidetes* [37]. Dai et al. demonstrated through ultra-high-performance liquid chromatography–mass spectrometry that the levels of *Lactobacillus*, *Streptococcus*, *Bacteroides*, and *Prevotella* were higher in tumor tissues compared to non-tumor tissues [38]. Pimentel-Nunes et al. demonstrated that GC tissues exhibited enrichment in *Firmicutes*, *Gemella*, and *Streptococcus*, alongside a reduction in *Proteobacteria* [39]. Zhou et al. found significant enrichments of *Streptococcus anginosus (Sa)* and *Streptococcus constellatus (Sc)* in the tumor tissues and feces of patients with intraepithelial neoplasia, as well as early and advanced GC [40]. Additionally, several studies consistently found an enrichment of *Lactobacillu* [41,42], *Streptococcus* [28,43], and *Bacteroidetes* [43,44,45] in GC. Furthermore, *Fusobacterium*, closely associated with CRC development, has recently been reported to be abnormally enriched in gastric adenocarcinoma [19,28,44,46].

Regarding changes in bacterial alpha diversity in GC, the observed results are not entirely consistent. Francisco et al. found a decreasing trend in bacterial diversity from non-atrophic gastritis (NAG) to intestinal metaplasia (IM) and then to intestinal-type gastric cancer [41,47,48,49,50,51]. However, there are some contradictory reports on this subject [46,50,52]. Olabisi et al. analyzed nearly 200 gastric mucosal samples and found no significant difference in microbial diversity but observed differences in microbial composition [46]. These conflicting results may stem from factors including ethnicity, diet, sequencing techniques, the diverse composition of study populations encompassing both *H. pylori*-positive and *H. pylori*-negative individuals, and the potential impact of previous GC surgery. However, gastric surgery can reduce the diversity of the gut microbiota in GC [53,54]. The composition of intestinal flora is also different in different stages of GC. Chen et al. found that *Collinsella*, *Blautia*, *Anaerostipes*, *Dorea*, and *Lachnospiraceae* expressed differently between early and advanced GC patients [55]. Additionally, the analysis of microbial communities in different parts of the stomach may also be a potential reason for heterogeneous results [56]. Yang et al.’s research indicates that there are differences in microbial composition and metabolic products between proximal and distal GC, even though there is no significant difference in species diversity and abundance [56]. Therefore, specific changes between different gastric microenvironments may help reveal the true relationship between gastric bacteria and the development of GC.

### 2.3. Epstein–Barr Virus and GC

Epstein–Barr virus (EBV) is a gamma herpesvirus, and some GCs are closely associated with EBV infection [1]. Studies have reported that patients with EBV-associated GC (EBVaGC) account for approximately 8.7–10% of GC patients [57]. EBV latent membrane protein 2A (LMP2A) is a transmembrane protein expressed by EBV and involved in EBVaGC. LMP2A can contribute to the phosphorylation of signal transducer and activator of transcription 3 (STAT3), activate the transcription of DNA methyltransferase 1 (DNMT1), induce extensive methylation of host genes, and affect the cell cycle and the microenvironment of GC [58]. EBVaGC has unique clinicopathological and molecular features, such as DNA hypermethylation, mutations in the PIK3CA gene, and high expression of programmed death receptor ligand 1 (PD-L1) [1]. Clinically, EBVaGC tends to be located in the proximal region, with a higher incidence in males than females [59], exhibits a relatively low rate of lymph node metastasis [60], and has a better prognosis [61]. Currently, the immunotherapy regimen for EBVaGC is similar to the other three GC subtypes. EBV infection is considered a potential biomarker for the immune therapeutic response, and the efficacy of ICB in EBVaGC patients is superior to that in unselected GC patients [62]. Nevertheless, EBVaGC patients still require precision treatment strategies in the future.

In addition, human immunodeficiency virus type 1 (HIV) is a retrovirus that predominantly infects human CD4+ T cells via the digestive tract. Recent studies have shown that HIV exposure is associated with a high risk of gastric cardia cancer [63]. However, the mechanism is unclear. HIV-infected individuals were more susceptible to active EBV infection [64]. Recently, Wahl et al. found that gut flora residents could increase the odds of HIV and EBV infection, as well as increase the density of HIV-infected CCR5+ CD4+ T cells [65]. These studies suggest that interactions between intestinal viruses, bacteria, and host immune cells may collectively influence the onset and progression of GC.

### 2.4. Fungi and GC

Similar to bacteria, fungi are an essential part of the human microbiome and play a crucial role in maintaining the delicate balance of microorganisms in the body [66]. Fungal sequences constitute a relatively small proportion of the microbial sequences found in tissue [67]. Whole-genome sequencing analysis of various tumor samples in TCGA revealed the presence of one fungal microbe per 10^4^ tumor cells [67]. Throughout the gastrointestinal tract, fungal DNA was relatively more abundant in the head and neck, colorectal, and gastric tissues, while it was less abundant in the esophagus [67]. In 2021, Zhong et al. utilized macrogenomic analysis to examine fungal changes in GC tumor tissues and adjacent non-cancerous tissues [68]. Their findings indicated a decrease in species richness, diversity, and evenness of fungal components during gastric carcinogenesis, along with significant alterations in fungal community structure. *Ascomycetes* emerged as the most enriched phylum in GC tissues, contrasting with decreased enrichment overall. Specifically, *Candida albicans*, *Clostridium* spp., *Staphylococcus aureus*, and *Clostridium quaticum* were identified as overcolonized at the species level in GC tissues. In 2022, Anders et al. demonstrated that elevated levels of *Candida* in GCs correlated with pro-inflammatory immune profiles. Additionally, *Candida* presence was predictive of metastatic disease and reduced cellular adhesion in colon cancers [67]. The study of fungi in GC is still in its early stages, and further exploration is needed to determine whether fungal dysbiosis is a cause or a consequence of GC (Table 1).

## 3. The Development of Microbial-Sequencing Technologies

Currently, most studies investigating microbiota changes associated with GC primarily involve obtaining gastric mucosal samples via surgical resection or biopsy. In addition to these methods, gastric fluid represents another potential source for studying the gastric microbiome. Given the proximity of the stomach’s downstream channel to the intestine, the development of GC could significantly impact the patient’s intestinal microbiota. Consequently, the analysis of fecal samples has emerged as a non-invasive and convenient research approach.

Advancements in high-throughput sequencing technology have revolutionized our understanding of the gastrointestinal microbiota. Microbiome research encompasses various “omics” methods, including culture-based methods, DNA sequencing, RNA sequencing, metabolomics, and proteomics. Culture-based methods, involving isolating and cultivating microorganisms, traditionally generate pure bacterial cultures. However, due to the challenges in culturing many microorganisms under laboratory conditions, this method cannot comprehensively capture microbial diversity [69]. DNA sequencing enables us to identify the species and genomes of microorganisms, while RNA sequencing reveals microbial gene expression activities. Metabolomics and proteomics provide insights into microbial metabolism and protein functions. These comprehensive approaches help us achieve a holistic understanding of the role and significance of the gastric microbiota within the gastric ecosystem.

Second-generation sequencing of the 16S rRNA gene, a widely employed analytical method in the field of microbiology, focuses on identifying the types of bacterial microorganisms and estimating their relative abundance [70]. Metagenomic techniques, on the other hand, allow for the calculation of the abundance of bacterial species in any given environment, revealing how the relative composition of microbial communities’ changes in response to various stimuli. Shotgun sequencing is the foundation of metagenomic sequencing, allowing for comprehensive analysis of entire microbial communities. Its applications are broad, providing detailed genomic information [71,72]. However, our understanding of how bacteria respond to environmental changes remains limited [73]. In recent years, third-generation sequencing technologies, exemplified by nanopore sequencing, have rapidly advanced. These technologies greatly aid in studying the heterogeneity within bacterial populations, facilitating the identification of rare bacterial subgroups [74,75].

However, regardless of the strategy employed, researchers face a challenge, which is the potential for misinterpretation due to contamination. Contaminants can be introduced at various stages, from sample collection to laboratory processing, and it is nearly impossible to eliminate them. To reduce the likelihood of misinterpretation, it is recommended to include “blank” samples in each experiment and use “bioinformatics” contamination filters provided in public databases. Furthermore, the results need to be interpreted cautiously because differences can arise from various factors, including different sequencing platforms; sequencing depths; the 16S rDNA target regions used for PCR amplification; DNA extraction tools; and the methods of sample collection, transportation, and storage. Variability in study populations, clinical conditions, and the diversity of metagenomic methods also influence result interpretation.

Although these methods contribute to our understanding of microbial composition, further research is needed to comprehend the precise role of gastric microbiota in disease development. Future work should emphasize functional studies, delving into the ecology and metabolic functions of microorganisms, thereby advancing clinical applications and therapeutic strategies.

## 4. The Pathogenic Mechanisms of Microbes in GC

**Figure 1:**
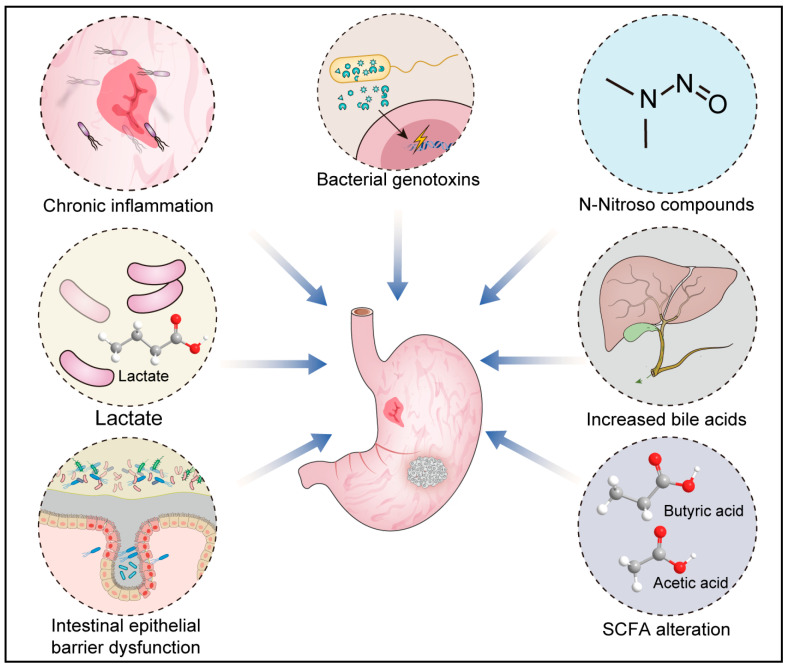
The relationship between intestinal microorganisms and gastric cancer.

Microorganisms are involved in carcinogenesis in both direct and indirect ways. Classical explanations of microbial carcinogenesis usually revolve around direct models of inflammatory dysregulation. However, contemporary perspectives on the role of microorganisms in cancer development have expanded to include their potential to influence a variety of cancer characteristics. Possible factors involved in microbial carcinogenesis include metabolites such as bile acids, choline, neurotransmitters, and SCFA; impaired intestinal mucosal barriers; altered immune responses; and bacterial genotoxins that induce DNA damage and genomic instability (Figure 1).

Microorganisms contribute to gastric cancer through several mechanisms. Pathogenic bacteria induce chronic inflammation, secrete genotoxins that destabilize DNA, alter short-chain fatty acid composition, and increase bile acid production. Dysbiosis in the gastric microbiome weakens the intestinal epithelial barrier, enhancing the adhesion of pathogens like *H. pylori*. Additionally, pathogenic bacteria increase N-nitroso compounds and utilize lactic acid produced by lactic acid bacteria to generate more harmful factors.

### 4.1. Chronic Inflammation

Chronic inflammation has been associated with the development of several malignant diseases, with a particularly strong correlation between *H. pylori* and GC. Several pathogenic mechanisms of *H. pylori* include altered host gene expression, infection-induced cell proliferation, elongation and loss of polarity of epithelial cells, degradation of cell–cell junctions, and decreased gastric acid secretion [76]. Although multiple “hits” are required for cancer induction, one of the most critical mechanisms may be the induction of chronic inflammation by *H. pylori* [75]. *H. pylori* infection is the classic initiator of chronic non-AG, AG, intestinal epithelial hyperplasia, dysplasia, and ultimately GC [75]. *H. pylori* infection induces inflammation through a variety of pathways, starting with the initially infected gastric epithelial cells and recruiting circulating immune cells to the site of infection. In patients with *H. pylori* infection, inflammatory factors, including IL-1, IL-6, IL-8, TNF-α, and NFκB, are significantly elevated [77,78]. Engevik et al. discovered that *F. nucleatum* secretes outer membrane vesicles (OMVs) to trigger TLR4 and NF-κB activation in colonic epithelial cells, leading to the initiation of downstream proinflammatory factors [79]. Notably, proinflammatory effects were absent in the context of an intact gut microbiota, suggesting the importance of the normal microecology [80].

### 4.2. Bacterial Genotoxins

Bacterial genotoxins are effectors that cause DNA damage by introducing single- and double-strand DNA breaks in the host cells [81]. Such DNA damage in somatic cells may cause genome damage, cell cycle arrest, and apoptosis; modulate the immune response; and launch carcinogenesis in the host [81]. For example, three genotoxins identified in *Escherichia coli* in CRC are involved in carcinogenesis, including cytolethal distending toxin (CDT), colibactin, and UshA [82]. It has been shown that *H. pylori* infection can also mediate genomic instability and altered cell polarity with carcinogenesis through the action of a range of bacterial virulence factors including, but not limited to, urease, vacuolating cytotoxin A (VacA), cag pathogenicity islands, cytotoxin-associated gene A (CagA), peptidoglycan outer membrane proteins (e.g., BabA, SabA, and OipA), and γ-glutamyl transpeptidase (GGT) [83]. *H. Pylori* strains with different virulence factor differences may end up with different clinical outcomes for *H. pylori*. The EBV-associated genes *LMP1*, *LMP2A*, *LMP2B*, *EBNA3C*, and *EBNA1* also contribute to altered cellular asymmetry [83]. Testing for microbial genotoxicity facilitates the identification of stronger risk factors for gastric disease.

### 4.3. Short-Chain Fatty Acid (SCFA) Alteration

SCFA are monocarboxylic acids with a chain length of C2 to C6, which are mainly produced by the fermentation of fiber components in food by intestinal flora, of which more than 95% are composed of acetic, propionic, and butyric acids [84]. SCFA are commonly used as energy substrates for colonocytes, stimulate mucus production, promote mucosal immunity and barrier function, and work together to maintain intestinal homeostasis [85]. Small amounts of SCFAs from the gut enter the systemic circulation and act as important signaling molecules [86]. *Bacteroides* provide most of the acetate and propionate, and *Firmicutes* is considered the main producer of butyrate [87]. Studies have shown that butyric acid and propionic acid induce apoptosis and necrosis to varying degrees in GC cells and cause cells to stagnate in the G2-M phase, which may have an inhibitory effect on GC [88]. Plasma levels of propionate and butyrate were significantly lower in GC patients compared to chronic superficial gastritis [89]. The presence of SCFA in the host’s digestive system may positively influence cancer treatment and slow down the cancerous process [86].

### 4.4. Increased Bile Acids

Bile acids and bile reflux are strongly associated with the development of gastric precancerous lesions and GC [90]. There are two major sites of bile acid biosynthesis: hepatocytes within the host and microorganisms in the gastrointestinal tract [91]. Host hepatocytes synthesize primary bile acids from cholesterol, and once these host-derived primary bile acids enter the gastrointestinal tract, the intestinal microbiota chemically modify them into secondary bile acids. These bile salt hydrolase-rich flora are included in the Gram-positive *Lactobacillus*, *Enterococcus*, *Clostridium* spp., and Gram-negative *Bacteroides* spp. bacteria and are also present in several bacterial strains (e.g., *L. plantarum*, *L. acidophilus*, *L. salivarius*, *C. perfringens*, etc.) [92]. Bile acids in the gut undergo a multistep metabolism by the intestinal flora, constituting a rich diversity of bile acid profiles. Bile acid also serves as a host factor that governs the composition of the cecal microbiota [93].

Bile acids promote tumor progression and telomerase activity in mice with GC, and this effect is dependent on the expression of c-Myc [94]. In addition, bile acids induce DNA damage, mutation, and reduce apoptotic capacity. *H. pylori* infection and exposure to bile acids may act synergistically to enhance the occurrence of GC. A Japanese study indicated that in patients with *H. pylori* infection, the severity of gastric mucosal atrophy and intestinal metaplasia intensifies with increasing concentrations of bile acids, also leading to an elevated risk of GC [95]. Another animal experiment indicates that in the presence of both iron deficiency and *H. pylori* infection, there is an increased production of deoxycholic acid [96]. Bile acid exposure contributes to the intracellular movement of cancer-associated protein (CagA), promoting gastric carcinogenesis [96]. In gastric juice, the interaction of bile acid and microbiome promotes gastric precancerous lesions via the STAT3 pathway [90,97].

### 4.5. Intestinal Epithelial Barrier Dysfunction

The mucus layer serves as a protective barrier, segregating the microbiota from direct contact with the intestinal epithelial lining and preventing an inflammatory response. SCFA produced by *Akkermansia muciniphila* enters the intestinal epithelial cells via the G protein-coupled receptor (GPCR), increasing tight junction proteins claudin-3 and occludin expression, aiding the intestinal barrier function [98,99,100]. *Bifidobacteria* reduce intestinal endotoxin formation and increase the production of tight junction proteins, thereby decreasing intestinal permeability and bacterial translocation [101]. Thus, during the development of GC, decreased probiotic colonization and imbalance of intestinal flora homeostasis weaken the integrity of the intestinal epithelial barrier, leading to increased intestinal permeability. Prior to direct contact with endothelial cells, *H. pylori* secretes proteases and phospholipases to degrade the mucus layer on the surface of the gastric mucosa, which enhances *H. pylori* adhesion [102]. OMVs released by *F. nucleatum* can also disrupt epithelial homeostasis by compromising the intestinal mucosal barrier [103].

### 4.6. N-Nitroso Compounds

The GC microbiota has increased nitrate reductase and nitrite reductase function compared to chronic gastritis [51]. Ferreira et al. noted that bacteria possessing nitrate reductase activity were strongly linked to higher gastric juice pH and positively correlated with increased N-nitrosamines concentrations [104]. A possible hypothesis is that during carcinogenesis, changes in the gastric mucosa lead to a decrease in gastric acid secretion, which allows the growth of bacteria capable of reducing nitrate to nitrite [51,105]. Nitrate reduction leads to the formation of N-nitroso compounds, which in turn promote epithelial cell mutagenesis, angiogenesis, and proto-oncogene expression, ultimately contributing to the development of GC [106,107]. *Escherichia coli*, *Enterococci*, Gram-positive *cocci*, and *Lactobacillus* are among the bacteria capable of converting nitrate to nitrite [108,109,110]. Certain species present in the oral cavity could also facilitate these reactions [110].

### 4.7. Lactate

Lactate can act as an energy source for oxidative cancer cells, hinder the function of T and NK cells, boost DNA-repair capabilities, and play a role in cell migration and resistance to chemotherapy [111,112,113,114]. An expanding population of lactic acid bacteria, such as *Streptococcus*, *Bifidobacterium*, *Lactobacillus*, *Lactococcus*, and various others, has been detected in patients with GC [42,52,115]. By employing isotopic labeling of lactic acid, Bourriaud and colleagues discovered that specific bacteria can utilize lactic acid to produce SCFA [116]. The most compelling in vivo support for the involvement of lactic acid-producing bacteria in GC stems from research conducted in the insulin-gastrin (INS-GAS) transgenic mouse model. Kvin et al. found that colonizing the stomach with a limited commensal microbiota (decreased *Clostridum* and *Bacteroides*, increased *Lactobacillus*) can emulate the promotion of neoplastic lesions in the INS-GAS mouse model of GC [117].

## 5. Influence of Gastrointestinal Microbes on GC Treatment

Recent research has increasingly emphasized the substantial impact of tumor microenvironment microorganisms and their metabolites on the efficacy of cancer therapy. Preclinical and clinical evidence indicates a robust connection between the gut microbiota and a wide range of chemotherapeutic agents such as oxaliplatin, irinotecan, gemcitabine, 5-fluorouracil, as well as immunotherapies, including PD-1/PD-L1 inhibitors and CTLA-4 inhibitors [118]. In the realm of chemotherapy and immunotherapy, gastrointestinal microorganisms can either directly or indirectly generate three distinct clinical outcomes: (i) enhancing drug effectiveness, (ii) hindering anticancer impacts, and (iii) influencing therapeutic side effects [119]. The management of these therapies is guided by a structured microbiota mechanistic framework called “TIMER,” representing translocation, immunomodulation, metabolism, enzyme degradation, diversity reduction, and ecological variation [120].

### 5.1. Chemotherapy

The gastrointestinal microbiota can intricately shape the response to chemotherapeutic agents, with instances such as the antitumor effects of 5-fluorocytosine and regafur (a precursor of 5-fluorouracil) being linked to derivatives of *Escherichia coli* [120]. Various microbiota exhibit diverse enzyme functions that have a substantial impact on both the efficacy and potential toxicity of chemotherapy [118]. Bacterial-derived nitroreductase plays a role in augmenting the activation of CB1954, a prodrug of gemcitabine, whereas the cytidine deaminase from Aspergillus origin may facilitate the inactivation of gemcitabine [120,121]. Butyrate was associated with enhanced activation of irinotecan [122]. Certain bacterial colonies displaying enzymatic beta-glucuronidase activity can transform the inactive form of irinotecan (SN38G) into its active form (SN38) within the gut, resulting in intestinal toxicity [123].

Oxaliplatin commonly induces peripheral neurotoxicity, affecting over 30% of patients. Studies show that the efficacy of Oxaliplatin is diminished in mice subjected to antibiotic interventions or raised in a sterile environment due to the absence of gut microbiota, leading to reduced inflammatory mediators and impaired ROS production by myeloid cells [124]. Furthermore, *Clostridium nucleatum* contributes to resistance against oxaliplatin and 5-fluorouracil by targeting TLR4 and MYD88, thereby activating autophagy and stimulating microRNA expression [125]. However, there is a mutual interaction between microbiota and chemotherapy, as chemotherapy can also result in drug toxicity and epithelial damage by disrupting the microbial balance and inducing metabolic changes [126].

### 5.2. Immunotherapy

Immunotherapies, including immune checkpoint blockade (ICB) treatments, are rapidly reshaping the standard of care for advanced gastric cancer patients by offering the potential for long-term disease control in certain individuals. Multiple studies have validated the pivotal role of the gastrointestinal microbiota in influencing the effectiveness and resistance of immune checkpoint blockade (ICB) therapies [127]. *Bifidobacterium* has the capacity to stimulate antigen-presenting cells, thus augmenting the effectiveness of PD-1/PD-L1 blockade therapies [128]. The effectiveness of CTLA-4 blockade therapy (Lpilimumab) was associated with specific T-cell responses facilitated by Bifidobacterium thetaiotaomicron or *Bifidobacterium fragilis* [129]. In addition, *Akkermansia muciniphila* affects the efficacy of the PD-1 antibody (Nivolumab) against epithelial tumors, is dependent on IL-12, and involves the recruitment of CCR9+ CXCR3+ CD4+ T lymphocytes [130]. However, *Mycobacterium methylis* has been shown to decrease TGFβ expression and suppress CD8+ tissue-resident memory T cells [47]. In addition, PD-L1 expression in tumors of GC patients was significantly correlated with *H. pylori* infection status [131]. *H. pylori* exposure inhibited CD4+ T cell proliferation and induced T cell apoptosis in gastric epithelial cells, and this effect was reversed by a PD-L1 antibody (Pembrolizumab), suggesting a potential candidate for ICB therapy [131,132,133]. The microbiota’s influence on the effectiveness of ICB may also be associated with its metabolic functions [134]. For example, inosine from *Bifidobacterium bifidum* enhances antitumor immunity [135]. In addition, SCFA originating from intestinal bacteria affects immune cell differentiation and function [136].

### 5.3. Radiotherapy

Radiotherapy is a type of cancer treatment based on high doses of radiation to kill cancer cells and shrink tumors and is considered a milestone in oncology. Approximately 50% of patients diagnosed with cancer would receive either aggressive or palliative radiation therapy [137]. In the mouse model, the augmentation of radiation’s anticancer effects was evident when Gram-positive bacteria were eradicated by antibiotics [138]. This effect was strictly dependent on a functioning immune system and was abrogated by sodium butyrate from Gram-positive bacteria [138]. Complete depletion of gut bacteria has also been shown to reduce the efficacy of radiation therapy in mouse models of breast cancer and melanoma, whereas depletion of gut fungi enhanced this efficacy due to an opposing effect on immune recruitment [139].

Through a multi-omics analysis, Guo and colleagues discovered that *Lachnospiraceae* and *Enterococcaceae*, along with bacteria-derived metabolites such as SCFA and tryptophan pathway members (I3A and KYNA), exhibited protective functions against radiation-induced mortality [140]. Abdominal radiation induces gut microbiota dysbiosis characterized by decreased diversity, primarily marked by a notable decline in aerobic and beneficial bacterial populations (e.g., *Lactobacilli* and *Bifidobacteria*) [141]. Dysbiosis of the intestinal flora aggravates radiation enteritis by weakening the function of the intestinal epithelial barrier and promoting the expression of inflammatory factors, thus exacerbating the development of enteritis. Derrien et al. showed that the clinical symptoms of radiotherapy were closely linked to intestinal flora imbalance, characterized by the enrichment of *Salmonella* and *Micrococcus* wartii and a decrease in the *Firmicutes* phylum [142].

## 6. Future Therapeutic Approaches Targeting the Microbiome

**Figure 2:**
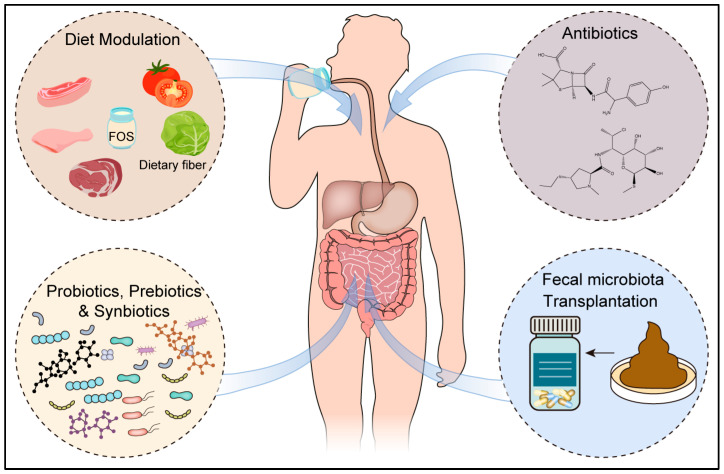
Emerging microbiome-based strategies in cancer therapy.

There is a growing interest in harnessing the microbiome to enhance cancer treatment outcomes. Researchers are investigating various approaches, including fecal microbial transplantation (FMT), probiotics, prebiotics, and other interventions, to restore and optimize the microbiome, thereby improving the efficacy of cancer treatment (Figure 2).

Key therapeutic approaches include dietary modulation, antibiotics as microbiome modulators, probiotics, prebiotics, synbiotics, and fecal microbiota transplantation. These interventions aim to enhance anti-tumor efficacy, reduce treatment-related side effects, and offer promising potential in improving cancer patient outcomes.

### 6.1. Diet

Tumor growth and survival rely on host-provided nutrition. Modifying dietary intake can potentially alter nutrient availability in the tumor microenvironment, offering a promising strategy for tumor control [143]. A high-fat diet (HFD) could stimulate intestinal stem cells and promote intestinal regeneration and oncogenesis [144]. Animal studies have shown that a diet rich in proteins (casein and whey), L-leucine, fish oil, and oligosaccharides can prevent Pseudomonas aeruginosa translocation, thus mitigating CTX-induced neutropenia [145]. Dietary fiber intake, including oligoisomaltose, oligofructose (FOS), and inulin, has been found to reduce irinotecan-associated toxicity by promoting intestinal butyrate production [146]. The ketogenic diet improved tumor control and survival during radiation therapy [147]. While there is a strong link between diet and gastrointestinal microbial diversity [136], it remains to be determined whether a universally beneficial dietary regimen exists that can simultaneously improve treatment efficacy and minimize toxicity.

### 6.2. Antibiotics Act as Modulators of Microbiota

Antibiotics are commonly used in solid cancer animal models to simulate the loss of microbial diversity, and evidence for the importance of the microbiome comes from probiotic therapy used to reverse the effects of antibiotics. Compared to mice receiving oxaliplatin treatment without antibiotics, those administered antibiotics alongside oxaliplatin displayed compromised cancer regression, decreased overall survival, and alleviated associated side effects [148].

While antibiotic treatment theoretically has the potential to eradicate bacterial populations linked to immune-related adverse events (irAEs), several clinical studies have noted diminished responses to immunotherapy in patients undergoing antibiotic treatment [149]. This can be attributed to the broad-spectrum antibiotics’ impact on the beneficial gut bacteria required for optimal outcomes in ICB treatment. Patients who refrained from using antibiotics within 42 days prior to starting ICB therapy experienced a 3.43-fold increase in overall survival compared to those who had used antibiotics within 60 days before ICB therapy [150]. This underscores the importance of the timing of antibiotic use.

### 6.3. Probiotics, Prebiotics, and Synbiotics

Probiotics are defined as living microorganisms that, when ingested in sufficient quantities, can provide health benefits to the body. Xiong et al. demonstrated that administering probiotics preoperatively to gastric cancer patients can lower postoperative inflammation levels and preserve the diversity and abundance of microorganisms after surgery [151]. Prebiotics are defined as indigestible dietary fibers fermented by intestinal bacteria [151] 148 and primarily have a beneficial impact on the gastrointestinal microbiota. Symbiotics consist of a combination of probiotics and prebiotics [152]. Research indicated that the supplementation of a probiotic mixture such as BIO-three, composed of *Bacillus*, *Butyricicoccus*, and *Faecalibacterium*, can mitigate oxaliplatin-induced intestinal damage in GC patients [148]. Postoperative supplementation of probiotics in GC can reduce complications, promote nutritional recovery, and improve prognosis [53,153]. Clinical studies have demonstrated that supplementing certain probiotics in conjunction with antibiotic treatment for *H. pylori* can improve treatment outcomes [154]. Furthermore, many studies have found that common prebiotics like inulin or oligofructose can increase the abundance of *Bifidobacteria*, *Lactobacilli*, and *Fecalibacterium*, assisting in cancer treatment [155,156]. Dietary inulin has also been shown to exert preventive effects on cancer metastasis by promoting the production of SCFA [157]. Additionally, the use of synbiotics as supplements has been found to ameliorate post-treatment symptoms in cancer patients, including nausea, vomiting, anorexia, diarrhea, and febrile neutropenia [158,159,160].

### 6.4. Fecal Microbiota Transplantation (FMT)

FMT is another effective method for restoring ecological balance and “normalizing” or “resetting” a healthy microbiota [161]. FMT has received FDA approval for the treatment of recurrent and refractory Clostridium difficile infection1 [162]. Early successes have been achieved by combining FMT with ICB therapy for refractory melanoma patients [163]. This approach involves transplanting fecal microbiota from cancer patients who respond to ICB into germ-free or antibiotic-treated mice, enhancing the anti-tumor effects of PD-1 blockade [130]. Additionally, FMT can reprogram the tumor microenvironment to overcome resistance to PD-1 antibody treatment [164]. Recently, a single-arm, open-label, investigator-initiated clinical trial explored the potential of overcoming resistance to anti-PD-1 therapies in gastrointestinal cancers by using FMT. Preliminary data from this trial suggest that combining FMT capsules with Nivolumab may alter the gut microbiota structure and potentially overcome resistance to anti-PD-1 therapies in the treatment of gastrointestinal cancers [165].

## 7. Conclusions and Prospects

GC remains one of the most common cancers in the world, with a relatively high mortality rate. Due to the heterogeneity of its course, its diagnosis and treatment are limited and difficult, associated with a reduced prognosis for patients. Therefore, it has become crucial to understand the mechanisms underlying the development and progression of this cancer, with particular emphasis on environmental and genetic factors and the role of the immune system. The gastrointestinal microbial genome is considered the second largest genome in the human body, and its relationship with disease has been a hot research topic. The recognition that microbial communities play an important role in gastrointestinal malignancies is growing. The intestinal microbial community is one of the most intensively studied microecological environments, with a bacterial load of 10^10^ to 10^12^ colony-forming units (CFU)/mL. Notably, the human stomach has a lower bacterial load of 10^2^ to 10^4^ CFU/mL compared to the gut [166]. Despite the exponential growth in the study of the microbiome, our understanding of the symbiotic relationship between bacteria and GC is embryonic. *H. pylori* constitutes a pivotal risk factor in the etiology of GC; however, the development of GC is intricately influenced by a combination of microbial factors, encompassing both *H. pylori* and non-*H. pylori* elements, as well as host and environmental factors. However, due to limited access to the gastric mucosa, as well as many factors and constitution of the patients themselves, the results of gastrointestinal microbial assays are often heterogeneous, thus greatly limiting their therapeutic potential. It is particularly important to develop new, standardized methods to examine the microbiome, which will allow a comparison of patient populations and results obtained, including between molecular, histopathological, and immunological GC subtypes. In recent years, microRNAs (miRNAs), represented by liquid biopsy, have demonstrated immense potential as novel biomarkers in cancer diagnosis [161].

The study of microbiota and tumors faces several limitations. For instance, establishing a direct causal link between specific microbial changes and cancer is challenging, as it is often difficult to determine whether these changes cause cancer or are a consequence of other factors, such as inflammation. The gut microbiota is highly diverse among individuals, complicating the generalization of findings. Changes are influenced by diet, geography, genetics, and the environment, making it hard to identify standardized cancer-associated patterns. While microbiota-targeted therapies show promise, translating laboratory discoveries into effective clinical treatments is still in its early stages, and further clinical trials are needed to confirm their efficacy and safety for patients. There are many questions that remain to be addressed. Many interdisciplinary studies are still needed to fully understand the mechanisms involved in the development and progression of GC in order to comprehensively analyze the composition of the microbiota and its impact on the human body, as well as to gain insights into the functioning of the patient’s immune system.

In the future, we need to pay attention to the changes of the intestinal flora from multiple perspectives and in an all-round way through more scientific and rational research methods so that we can thoroughly and clearly understand the causes and results of the relationship between GC and intestinal flora, and not just confine ourselves to its correlation. On the one hand, we can accurately and scientifically transplant the needed flora among fecal bacteria and target dietary habits to combat harmful intestinal metabolites, bacterial products, and bacterial toxins; on the other hand, the low survival rate of GC is usually attributed to late diagnosis, and targeting the monitoring of patients who meet these criteria can increase the probability of the early detection of GC.

## Figures and Tables

**Table 1:** Study on intestinal microorganisms of gastric cancer.

Time	Region	Key Results	Method	Sample Size	Sample Type	References
2023	China	*Hp* increased significantly in peritumoral microhabitat of patients with good prognoses	16S rRNA	132 GC	Tissues	[20]
2023	Japan	In GC patients treated with *Hp* eradication, the average abundance of *Unclassified Oxalobacteraceae*, *Capnocytophaga*, and *Haemophilus* increased	16S rRNA	8 EGC vs. 9 NC	Tissues	[26]
2023	China	*Helicobacter* and *Lysobacter* were notably more abundant in normal tissues, whereas *Pseudomonas* was more prevalent in tumor tissues	RNA-Seq	727 GC	Tissues	[32]
2022	China	Gastric surgery can reduce the diversity of the gut microbiota in GC	16S rRNA	100 GC	Feces	[54]
2022	Korea	Gastric mucosal microbiota in GC patients showed reduced diversity and increased abundance of *Actinobacteria*, *Bacteroidetes*, and *Firmicutes*	16S rRNA	45 GC vs. 92 HC	Tissues	[49]
2022	Korea	The microbial diversity continuously decreased continuously from gastritis to GC	16S rRNA	88 GC	Gastric juice	[35]
2022	Singapore	GC development was marked by increased *Proteobacteria* and decreased *Bacteroidetes*	16S rRNA	89 (43 GC vs. others)	Tissues	[37]
2022	China	GC patients had significantly lower levels of *Faecalibacterium*, *Bifidobacterium*, and *Subdoligranulum*, and higher levels of *Enterococcus*, *Streptococcus*, and *Bacteroides*, compared to healthy individuals	16S rRNA	30 GC vs. 30 Normal	Feces	[43]
2022	China	Microbial composition and metabolic products differ between proximal and distal GC, though species diversity and abundance remain similar	16S rRNA	29 GC vs. NT	Tissues	[56]
2022	USA	High *Candida* levels were associated with pro-inflammatory immune pathways	External ITS sequencing	321 (from TCGA)	Tissues	[67]
2022	China	*Streptococcus anginosus* and *Streptococcus constellatus* were more common in GC tumor tissues	16S rRNA	1043 GC	Tissues and feces	[40]
2022	China	The microbial diversity of GC microbiota was reduced	16S rRNA	53 GC vs. 30 CG	Tissues	[47]
2022	China	The composition of intestinal flora was different in different stages of GC	16S rRNA	226 GC	Feces	[55]
2021	China	The abundance of *Lactobacillus*, *Streptococcus*, *Bacteroides*, and *Prevotella* was increased in tumor tissues compared to non-tumor tissues	16S rRNA	37 GC vs. NT	Tissues	[38]
2021	Portugal	GC tissues were enriched with *Firmicutes*, *Gemella*, and *Streptococcus*, while *Proteobacteria* were reduced	16S rRNA	77 GC	Tissues	[39]
2021	China	The species richness, diversity, and evenness of fungal components tended to decrease with gastric carcinogenesis, and the fungal community structure changed significantly. *Albicans* may be a biomarker for GC	ITS rDNA gene analysis	45 GC	Tissues	[68]
2020	USA/Japan	The overall bacterial alpha diversity metrics in the control group was higher than the cancer groups	16S rRNA	48 GC and 120 NC	Tissues	[50]
2020	HK	Eradication of *Hp* treatment can lead to an increase in bacterial diversity	16S rRNA	202 GC	Tissues	[25]
2020	China	Bacterial diversity and phyla Armatimonadetes, *Chloroflexi*, *Elusimicrobia*, *Nitrospirae*, *Planctomycetes*, *Verrucomicrobia*, and WS3 decreased from CG to GC, while *Actinobacteria*, *Bacteroides*, *Firmicutes*, *Fusobacteria*, SR1, and TM7 increased in IN and GC	16S rRNA	132 (29 GC vs. others)	Tissues	[44]
2020	China	In advanced gastric lesion patients, *Helicobacter* showed strong avoidance of co-occurrence with *Fusobacterium*, *Neisseria*, *Prevotella*, *Veillonella*, and *Rothia*	16S rRNA	115 GC	Tissues and feces	[30]
2019	China	Bacterial richness decreased in peritumoral and tumoral microhabitats, and the correlation network of abundant gastric bacteria was simplified in the tumoral microhabitat	16S rRNA	276 GC	Tissues	[28]
2018	China	*Hp* abundance was lower in tumor tissues compared to adjacent non-tumor tissues	16S rRNA	11 GC vs. 16 NC	Tissues	[19]
2018	Portugal	Patients with GC exhibit a dysbiotic microbial community with genotoxic potential	16S rRNA	54 GC and 81 CG	Tissues	[51]
2017	USA	*Hp* dominated the non-malignant gastric tissue microbiota in many GC patients	16S rRNA	160 GC	Tissues	[23]
2017	Australia	Increased richness and phylogenetic diversity in GC	16S rRNA	12 GC vs. 20 FD	Tissues	[52]
2017	Sweden	*Hp* abundance was positively correlated with *Campylobacter*, *Deinococcus*, and *Sulfurospirillum*	Metatranscriptomic RNA sequencing	149 GC	Tissues	[31]
2017	China	*P. stomatis*, *D. pneumosintes*, *S. exigua*, *P. micra*, and *S. anginosus* may play important roles in GC progression	16S rRNA	200 (GC, AG, IM, SG)	Tissues	[46]
2016	China	The microbiota structure in GC was more diverse	16S rRNA	12 GC	Tissues	[27]
2014	Mexico	Bacterial diversity decreases from NAG to IM to intestinal-type GC	G3 PhyloChip	5 GC	Tissues	[41]
2010	USA	*Hp* GC showed increased levels of *Proteobacteria*, *Firmicutes*, *Actinobacteria*, and *Bacteroidetes*	16S rRNA	12 GC	Tissues	[24]
2006	USA	*Hp* presence did not affect gastric community composition. The gastric flora comprises 128 diverse phylotypes	16S rRNA	23 GC	Tissues	[9]

Abbreviations: *Hp*: *H. pylori*; AG: atrophic gastritis; CG: chronic gastritis; Controls: chronic gastritis or intestinal metaplasia; FD: functional dyspepsia; GC: gastric cancer; HC: healthy controls; IM: intestinal metaplasia; NAG: non-atrophic gastric; NC: non-tumor tissues; SG: superficial gastritis.
